# Baseline Gut Microbiota Was Associated with Long-Term Immune Response at One Year Following Three Doses of BNT162b2

**DOI:** 10.3390/vaccines12080916

**Published:** 2024-08-14

**Authors:** Li-Na Zhang, Jing-Tong Tan, Ho-Yu Ng, Yun-Shi Liao, Rui-Qi Zhang, Kwok-Hung Chan, Ivan Fan-Ngai Hung, Tommy Tsan-Yuk Lam, Ka-Shing Cheung

**Affiliations:** 1 Department of Medicine, School of Clinical Medicine, The University of Hong Kong, Queen Mary Hospital, Hong Kong, Chinatjt97@connect.hku.hk (J.-T.T.); zhangrq@hku.hk (R.-Q.Z.); ivanhung@hku.hk (I.F.-N.H.); 2 School of Clinical Medicine, The University of Hong Kong, Hong Kong, China; nghoyu@connect.hku.hk; 3 State Key Laboratory of Emerging Infectious Diseases, School of Public Health, The University of Hong Kong, Hong Kong, China; yunshi.liao@c2i.hk (Y.-S.L.); ttylam@hku.hk (T.T.-Y.L.); 4 Centre for Immunology & Infection Limited, 17W Hong Kong Science & Technology Parks, Hong Kong, China; 5 Department of Microbiology, School of Clinical Medicine, The University of Hong Kong, Queen Mary Hospital, Hong Kong, China; chankh2@hku.hk; 6 Department of Medicine, The University of Hong Kong-Shenzhen Hospital, Shenzhen 518000, China

**Keywords:** gastrointestinal microbiome, immunogenicity, vaccine, COVID-19

## Abstract

Background: This study explored neutralizing IgG antibody levels against COVID-19 decline over time post-vaccination. We conducted this prospective cohort study to investigate the function of gut microbiota in the host immune response following three doses of BNT162b2. Methods: Subjects who received three doses of BNT162b2 were recruited from three centers in Hong Kong. Blood samples were obtained before the first dose and at the one-year timepoint for IgG ELISA to determine the level of neutralizing antibody (NAb). The primary outcome was a high immune response (NAb > 600 AU/mL). We performed shotgun DNA metagenomic sequencing on baseline fecal samples to identify bacterial species and metabolic pathways associated with high immune response using linear discriminant analysis effect size analysis. Results: A total of 125 subjects were recruited (median age: 52 years [IQR: 46.2–59.0]; male: 43 [34.4%]), and 20 were regarded as low responders at the one-year timepoint. *Streptococcus parasanguinis* (log_10_LDA score = 2.38, *p* = 0.003; relative abundance of 2.97 × 10^−5^ vs. 0.03%, *p* = 0.001), *Bacteroides stercoris* (log_10_LDA score = 4.29, *p* = 0.024; relative abundance of 0.14% vs. 2.40%, *p* = 0.014) and *Haemophilus parainfluenzae* (log_10_LDA score = 2.15, *p* = 0.022; relative abundance of 0.01% vs. 0, *p* = 0.010) were enriched in low responders. *Bifidobacterium pseudocatenulatum* (log_10_LDA score = 2.99, *p* = 0.048; relative abundance of 0.09% vs. 0.36%, *p* = 0.049) and *Clostridium leptum* (log_10_LDA score = 2.38, *p* = 0.014; relative abundance of 1.2 × 10^−5^% vs. 0, *p* = 0.044) were enriched in high responders. *S. parasanguinis* was negatively correlated with the superpathway of pyrimidine ribonucleotides de novo biosynthesis (log_10_LDA score = 2.63), which contributes to inflammation and antibody production. *H. parainfluenzae* was positively correlated with pathways related to anti-inflammatory processes, including the superpathway of histidine, purine, and pyrimidine biosynthesis (log_10_LDA score = 2.14). Conclusion: Among three-dose BNT162b2 recipients, *S. parasanguinis*, *B. stercoris* and *H. parainfluenzae* were associated with poorer immunogenicity at one year, while *B. pseudocatenulatum* and *C. leptum* was associated with a better response.

## 1. Introduction

Since it started spreading in early 2020, the coronavirus disease 2019 (COVID-19) has infected over 7 billion people and caused a huge mortality, exceeding 7 million [1]. As one of the most effective methods against severe infection, vaccines have been administered to 67% of the global population with at least one dose [1]. However, recent studies have revealed that the antibodies induced by vaccination have declined over time [2,3]. Therefore, booster doses of vaccines have been employed to enhance the durability of antibodies [4]. In the latest recommendation, for BNT162b2, a third dose of 0.3 mL was advised, with a minimum interval of five months from the previous dose [5].

The immune responses induced by vaccines vary greatly among individuals due to various factors, including age, vaccination history, comorbidities (e.g., diabetes mellitus [DM]) and microbiota composition [6,7]. The association between microbiota and immunity has been continuously reported, and its role in vaccine immunogenicity has gradually been revealed [8,9,10]. Commensal microbiota can interact with pattern-recognition receptors (PRRs) [11] to influence the activities of antigen-presenting cells (APCs) and produce immunoregulatory metabolites, including flagellin [12], lipopolysaccharides (LPS) [13], peptidoglycans [14], short-chain fatty acids (SCFAs) [15] and secondary bile acids [16]. These bioactive substances can further act as natural immune adjuvants and interact with Toll-like receptors (TLRs) and nucleotide-binding oligomerization domain-containing protein 2(NOD2) to regulate the function of various immune cells, including dendritic cells, macrophages and neutrophils [6,17]. A randomized clinical trial found that the oral administration of *Lactobacillus fermentum (CECT5716)* was positively associated with increased immunogenicity to the Influenza H1N1 vaccine, likely due to the enhanced population of T helper cells and increased levels of neutralizing immunoglobulin A (IgA) antibodies [18]. Similarly, the treatment of BALB/c mice with *Lactobacillus rhamnosus* (CRL1505) can enhance the immune response to respiratory syncytial virus infection by increasing the secretion of interferon-γ and interleukin [19]. One study demonstrated that the pre-use of broad-spectrum antibiotics dampened gut bacteria diversity and dramatically reduced neutralizing immunoglobulin G (IgG) and IgA levels against the Influenza H1N1 virus after vaccination in certain groups of subjects [16].

An increasing amount of evidence suggests a potential correlation between microbiota composition and the immune response against COVID-19 vaccines [10]. One study found that a high abundance of the genus *Parasutterella* at baseline was associated with high antibody levels in ChAdOx1 recipients [20]. Further research on subjects receiving two doses of vaccination suggested that *Bifidobacterium adolescentis* was correlated with a high immune response in the CoronaVac group [21]. Similar analyses have reported a positive correlation between the abundance of *Collinsella aerofaciens, Fusicatenibacter saccharivorans, Eubacterium ramulus*, and *Veillonella dispar* and a high immune response, as well as between the enrichment of *Lawsonibacter asaccharolyticus* and a low immune response. An elevated level of SCFAs was demonstrated to be associated with high vaccine response [22].

Current studies are limited to only two doses of vaccination and have relatively short follow-up periods. One study investigated the correlation between the baseline microbiota composition and vaccine response towards two-dose vaccination with one-month follow-up [21]. Another study included two doses of BBIBP-CorV subjects with 42-day follow-up [22]. However, boosters are commonly administered nowadays due to the known decline in antibody levels. In light of this, our study aims to explore the association between baseline microbiota composition and long-term vaccine immunogenicity following three doses of BNT162b2 over a one-year follow-up period.

## 2. Methods

### 2.1. Study Design and Subjects

This was a prospective cohort study. Subjects who had received a total of three doses of the BNT162b2 [23] (COMIRNATYTM COVID-19 mRNA Vaccine, Pfizer-BioNTech, Mainz, Germany) vaccination were recruited from three different centers in Hong Kong: Sun Yat Sen Memorial Park Sports Centre, Ap Lei Chau HKU Vaccination Centre, and Queen Mary Hospital. Subjects received their first two intramuscular doses (0.3 mL per dose) three weeks apart, followed by a booster administered at least six months after the second dose.

Subjects were excluded based on the following criteria: (i) under 18 years of age; (ii) inflammatory bowel diseases (IBD); (iii) immunocompromised status (including those using immunosuppressives/chemotherapy, post-transplantation status, and medical conditions such as cancer, hematological, rheumatological, and autoimmune diseases); (iv) prior use of probiotics, symbiotics and postbiotics within 12 months; (v) prior history of COVID-19; (vi) COVID-19 during the course of this study; (vii) subjects receiving the third dose within 84 days of the one-year timepoint. Subjects who received the booster less than 84 days before the one-year timepoint were excluded because antibody levels typically peak at approximately 84 days after vaccination [24,25]. Subjects with previous COVID-19 or with existing neutralizing antibodies against severe acute respiratory syndrome coronavirus 2 (SARS-CoV-2) were identified as having infection history.

### 2.2. Collection of Demographics, Anthropometrics and Blood Samples

Baseline demographic data, including age and sex, DM or pre-DM, were gathered. Additionally, information on medication use, including proton pump inhibitor (PPI), antibiotics, probiotics, symbiotics and postbiotics for 14 days or more within the past year was collected. Blood samples were obtained before the first vaccination and at the one-year timepoint after the first dose.

Vaccine immunogenicity was determined by neutralizing antibody (NAb) levels against the SARS-CoV-2 receptor-binding domain (RBD) based on IgG Enzyme-linked immunosorbent assay (ELISA) using a new version of the iFlash-2019-nCoV NAb kit (chemiluminescent microparticle immunoassay; Shenzhen YHLO Biotech Co., Ltd., Shenzhen, China) in this study. The NAb test served as a surrogate marker of vaccination effectiveness, indicating the protective power of antibodies against COVID-19 infection [26]. In brief, the serum sample and the reagent pack were placed according to the manufacturer’s instruction. The reagent pack contained RBD antigen (30KD)-coated paramagnetic microparticles and an acridinium ester-labeled ACE2 conjugate of the virus. Then, the iFlash system was activated, and a calibration curve was produced based on the signals of the chemiluminescent reaction. An NAb result of 15 AU/mL or more was considered seropositive, while the maximum measurable result was 800 AU/mL [27].

### 2.3. Shotgun Metagenomic Sequencing of Stool Samples

Baseline stool samples were collected from 125 subjects within one week before the first dose of vaccination. Sixty-six (52.8%) of these subjects also had stool samples collected at one year after first dose. The stool samples were delivered to the laboratory (kept at −80 °C) within 48 h. Subjects conducted the collection process in accordance with the manufacturer’s instruction (OMNIgene·GUT|OM-200, DNA Genotek Inc., Ottawa, ON, Canada) [28]. In summary, subjects were required to place a small amount of fresh stool into a yellow tube, level the sample with the provided spatula, and then vigorously shake the tube for over 30 s until the stool was mostly dissolved in the liquid, leaving only a small number of particles undissolved.

Genomic DNA was extracted using the Qiagen QIAamp DNA Stool Mini Kit (Qiagen, Hilden, Germany). The extracted DNA then underwent library construction with a Nextera DNA Library Prep Kit (Illumina, San Diego, CA, USA), a process that included fragmentation, adapter sequence addition, PCR amplification, and purification. After the library preparation was completed, its quality was evaluated using a Qubit fluorometer (Thermo Fisher Scientific, Waltham, MA, USA) and a bioanalyzer (Agilent Technologies, Santa Clara, CA, USA). Once the quality was confirmed, high-throughput sequencing was carried out on the Illumina NovaSeq 6000 platform, producing paired-end reads of 150 bp.

### 2.4. Primary Outcome of Interest

The primary outcome of interest was high immune response at the one-year timepoint. We defined subjects with neutralizing antibody (NAb) levels exceeding 600 AU/mL at one year as high responders, because NAb levels below 600 AU/mL have been reported to correlate with mortality due to breakthrough infection [29].

### 2.5. Bioinformatics Analysis

Raw reads from next-generation sequencing were processed using fastp v0.20.12 [30] to remove adapters and perform quality control. Subsequently, host sequences were eliminated using Bowtie2 [31] by aligning the reads to the human reference genome GRCh38.p13. Bacterial species identification for each sample was conducted using MetaPhlAn (v3.0) [32], and the abundance of bacterial metabolic pathways was determined using HUMAnN (v3.0) based on the gene ontology (GO) database [33]. Statistical analysis in this study was carried out with R statistical software (version 4.3.2). Alpha-diversity, measured by species richness, Shannon, and Simpson indices, was calculated with the “vegan” package. The difference between groups was compared using the Wilcoxon signed-rank test. Beta-diversity was presented by Bray–Curtis compositional dissimilarity and the non-metric multidimensional scaling (NMDS) method. Permutational multivariate analysis of variance (PERMANOVA) was used to calculate the difference of beta diversity between groups. Putative bacterial species and metabolic pathways were identified by the linear discriminant analysis effect size (LefSe, version 1.1.2) method. Species and metabolic pathways with an absolute value of linear discriminant analysis (LDA) score greater than or equal to 2 were selected [34]. Bacterial species with a median relative abundance of zero in either the low responders’ or high responders’ group were considered zero-inflated and excluded from subsequent analysis.

### 2.6. Statistical Analysis

Continuous variables were described by median and interquartile range (IQR), while categorical variables were presented in the form of count and ratio. Kolmogorov–Smirnov test was used to assess the normality of the data distribution. A *p*-value less than 0.05 indicated the data were not normally distributed. The Mann–Whitney U test was used to compare continuous variables between groups. For categorical variables in demographic data, either the Chi-square test or Fisher’s exact test was utilized. The dynamic change of microbiota composition between baseline and one year after the vaccination was measured by Jensen–Shannon distance (JSD) metrics [35]. A JSD value ≥ 0.4 indicates large variation, 0.15 ≤ JSD value < 0.4 indicates moderate variation, and JSD value < 0.15 indicates minimal variation. The correlation between bacterial species and metabolic pathways was calculated using Spearman’s correlation analysis and visualized with a heatmap. *p*-value was adjust by the false discovery rate (FDR) method [36]. Univariate and multivariable logistic regression models were used to identify clinical factors and bacterial species related to high immune response, as well as to calculate the odds ratios (OR), adjusted odds ratios (aOR) and *p*-values. We divided individuals having a relative abundance of a species in the top 50% of the population (i.e., above the median) into a high-abundance group.

Sensitivity analysis was performed by excluding subjects with prior use of antibiotics.

A two-sided *p*-value of less than 0.05 was deemed to indicate statistical significance.

## 3. Results

### 3.1. Baseline Characteristics

A total of 125 eligible subjects who received the complete course of three doses of BNT162b2 vaccination were enrolled in this study. As shown in Table 1, the cohort had a median age of 52 years (IQR: 46.2–59.0), including 43 (34.4%) males and 82 (65.6%) females. Among the subjects, 50 (40.0%) had DM or pre-DM, 18 (17.1%) had taken PPIs, and 5 (4.0%) had taken antibiotics for 14 days or more within the past year.

A total of 20 (16%) subjects with an NAb level less than 600 AU/mL (range: 53.80–594.47 AU/mL) were categorized into the low immune response group, while 105 (84%) participants with an NAb level over 600 AU/mL (range: 605.38–800.00 AU/mL) were categorized into the high immune-response group. There were more male subjects in the low immune-response group than in the high immune-response group (55.0% vs. 30.5%, *p* = 0.042) (Table 1).

Five subjects with prior use of antibiotics were excluded, and the baseline characteristics remained similar (Table S1).

### 3.2. Baseline Microbiota Composition Was Correlated with Vaccine Immunogenicity at One Year Following Three Doses of BNT162b2

**Figure 1:**
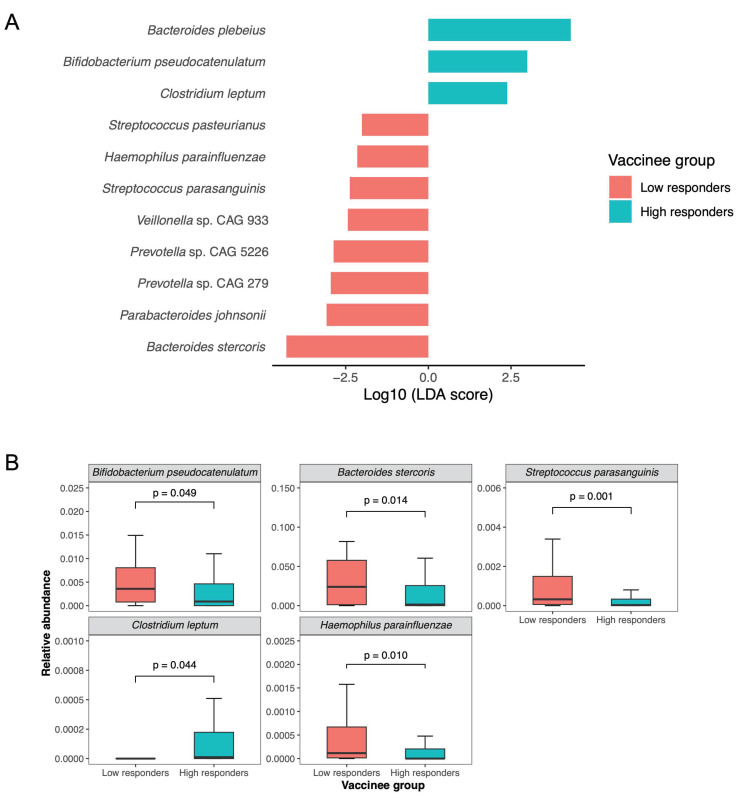
Baseline gut microbiota composition in low and high immune response group one year following three doses of BNT162b2. (**A**) Bacterial species enriched in low and high immune response groups identified by LEfSe analysis. (**B**) Relative abundance comparison of putative species (not zero-inflated) in low- and high-response groups.

There was no significant difference in alpha diversity (including Shannon index, Simpson index, and richness; all *p* > 0.05, Figure S2) and beta diversity (*p* = 0.174, Figure S2) between the low and high immune response groups. In the low responders, eight species were enriched based on LefSe analysis (Figure 1). Three of the species were not zero-inflated, namely *Streptococcus parasanguinis* (log_10_LDA score = 2.38, *p* = 0.003; relative abundance of 2.97 × 10^−5^ vs. 0.03%, *p* = 0.001), *Bacteroides stercoris* (log_10_LDA score = 4.29, *p* = 0.024; relative abundance of 0.14% vs. 2.40%, *p* = 0.014) and *Haemophilus parainfluenzae* (log_10_LDA score = 2.15, *p* = 0.022; relative abundance of 0.01% vs. 0, *p* = 0.010). Three bacterial species were found to be abundant in high responders, and among them, *Bifidobacterium pseudocatenulatum* (log_10_LDA score = 2.99, *p* = 0.048; relative abundance of 0.09% vs. 0.36%, *p* = 0.049) and *Clostridium leptum* (log_10_LDA score = 2.38, *p* = 0.014; relative abundance of 1.2 × 10^−5^% vs. 0, *p* = 0.044) were not zero-inflated. In the sensitivity analysis excluding the five subjects with prior antibiotic use, four bacterial species (*Clostridium leptum*, *Bacteroides stercoris*, *Streptococcus parasanguinis*, and *Haemophilus parainfluenzae*) remained significant (Figure S3A).

Seventy-eight species with a median relative abundance greater than 0 were identified at baseline, and 79 species were identified at the one-year timepoint (Figure S4). The JSD value for microbiota composition between baseline and one year was 0.11, indicating minimal variation. There were no significant differences in alpha diversity (including Shannon index, Simpson index, and richness; all *p* > 0.05, Figure S5A) or beta diversity (*p* = 0.982, Figure S5B) between the two timepoints. There was also no significant difference in the distribution of the putative species between the two timepoints (Figure S6).

In the multivariable analysis, high abundance of *Streptococcus parasanguinis* was associated with a lower odds of high vaccine immune response (aOR:0.14, 95% CI: 0.03–0.60), while high abundance of *Clostridium leptum* was associated with higher odds of high vaccine immune response (aOR:12.2, 95% CI: 1.73–273) (Table 2).

### 3.3. The Correlation between Putative Bacterial Species and Metabolic Pathways

Sixteen metabolic pathways were enriched in low responders (Figure S7), which mainly correlated to amnio acid and bioactive substance synthesis, including histidine, L-methionine, butyrate synthesis, etc. Three metabolic pathways were enriched in high responders, including folate transformation II pathway (log_10_LDA score = 2.43, *p* = 0.039), thiamine diphosphate salvage II pathway (log_10_LDA score = 2.44, *p* = 0.022) and superpathway of pyrimidine ribonucleotides de novo biosynthesis (log_10_LDA score = 2.63, *p* = 0.001). These pathways belonged to superclasses (Table S2) including ‘Biosynthesis’, ‘Generation of Precursor Metabolites and Energy’, ‘Degradation/Utilization/Assimilation’ and ‘Superpathways’ according to the MetaCyc database. In the sensitivity analysis excluding the five subjects with prior antibiotic use, all these pathways identified above continued to be significant (Figure S3B).

**Figure 2:**
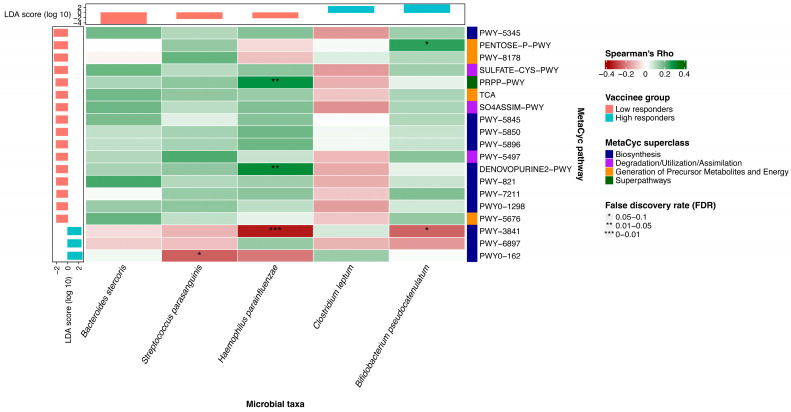
Correlation between baseline bacterial species and metabolic pathways using Spearman correlation analysis. Heatmap depicting the association between the relative abundances of microbial species and metabolic pathways, as well as their enrichment in groups of low and high responders. The *p*-values have been adjusted for false discovery rate (FDR). An asterisk indicates an FDR in the range of 0.05–0.1; two asterisks represent an FDR in the range of 0.01–0.05; three asterisks indicate an FDR in the range of 0–0.01. An FDR below 0.1 is considered statistically significant.

We found that the abundance of *Haemophilus parainfluenzae* was positively correlated with pathways related to anti-inflammatory processes, including the superpathway of histidine, purine, and pyrimidine biosynthesis (r = 0.29; *p* = 0.001) and the superpathway of purine nucleotides de novo biosynthesis II (r = 0.30; *p* < 0.001) (Figure 2). Its abundance was negatively correlated with folate transformation II pathway (r = −0.35; *p* < 0.001). The abundance of *Bifidobacterium pseudocatenulatum* was positively correlated with pentose phosphate pathway (r = 0.25; *p* = 0.004) and negatively correlated with folate transformation II pathway (r = −0.24, *p* = 0.006). The abundance of *Streptococcus parasanguinis* was negatively correlated with superpathway of pyrimidine ribonucleotides de novo biosynthesis (r = −0.25; *p* = 0.005). This pathway exerts pro-inflammatory function on various immune cells.

## 4. Discussion

This study represents the first prospective investigation into the association between baseline gut microbiota composition and vaccine immunogenicity following three doses of BNT162b2 with a one-year follow-up. We observed that the high relative abundance of *Bifidobacterium pseudocatenulatum* and *Clostridium leptum* was positively correlated with a high immune response, while the high relative abundance of *Bacteroides stercoris*, *Streptococcus parasanguinis* and *Haemophilus parainfluenzae* was positively associated with a low immune response. We identified sixteen metabolic pathways enriched in low immune responders, primarily associated with amino acid and bioactive substance synthesis, including histidine, L-methionine, and butyrate, among others. In contrast, three metabolic pathways were abundant in high responders and were related to vitamin synthesis. Most of these pathways exhibited significant correlations with *Streptococcus parasanguinis*, *Haemophilus parainfluenzae* and *Bifidobacterium pseudocatenulatum.*

Although the dominant bacterial species are critical to the host’s internal environment, species of low abundance may also exert important influences on the host. The distribution of these species of low abundance can influence the bioactivities of the dominant species, and their metabolites can also affect the immunity of the host. Several studies demonstrated the association between species of low abundance and diseases, such as *Methanobrevibacter smithii* (relative abundance ranging between 0.1% and 1%) and periodontitis, and *Fusobacterium nucleatum* (relative abundance of <1%) and colon cancer [37].

*Bifidobacterium pseudocatenulatum* belongs to the *Bifidobacterium* genus, which is well known for its probiotic properties. The administration of *Bifidobacterium pseudocatenulatum* has been shown to reverse immune suppression induced by a high-fat diet [38]. It facilitates the production of pro-inflammatory cytokines such as tumor necrosis factor-α (TNF-α) and interleukin 4 (IL-4), while downregulating anti-inflammatory factors including interleukin 10 (IL-10), interleukin 6 (IL-6), and monocyte chemoattractant protein-1 (MCP-1). This modulation occurs through its influence on macrophages, dendritic cells (DCs), and T cells [38,39,40]. Furthermore, *Bifidobacterium pseudocatenulatum* is capable of producing acetate [41], which has been shown to activate B cells and enhance the production of IgA via G-Protein Coupled Receptor 43 (GPR43) [42]. Additionally, acetate is known to enhance the function of CD8+ T cells by supporting their glycolytic activities [43] and to bolster the function of CD4+ T cells through Toll-like receptor 2 (TLR2) signaling [44]. *Clostridium leptum* was previously reported to be positively correlated with IgG production [45], and with neutrophil and natural killer cell counts [46]. *Clostridium leptum* can convert primary bile acids to secondary bile acids in the gut [47,48], which facilitates the innate immune response by interacting with G protein-coupled bile acid receptor 1 (GPBAR1) and Farnesoid-X-Receptor (FXR) [49].

On the other hand, *Streptococcus parasanguinis* may exhibit anti-inflammatory properties by regulating innate immune cells to produce a range of anti-inflammatory cytokines, notably IL-10, which has been reported to facilitate the differentiation of induced T regulatory (iTreg) cells [50,51]. As for *Bacteroides stercoris*, its abundance has been reported to positively correlate with the expression of the V-domain Ig suppressor of T cell activation (VISTA) gene, leading to the activation of Treg cells. Additionally, *Bacteroides stercoris* was reported to produce butyrate to inhibit histone deacetylase (HDAC) and maintain the function of Treg cells [52]. *Bacteroides stercoris* has also been found to be negatively associated with ulcerative colitis activity [53,54]. These functions of the species may explain their correlation with immune response. *Haemophilus parainfluenzae* is an opportunistic bacterium that is abundant in cases of severe infection [55].

It was reported that the decreased relative abundance of *Bacteroides* was positively correlated with a higher immune response to the rotavirus vaccine, which might be correlated with the variation in LPS component in the bacteria and results in immune inhibition in the host [56]. As a well-known kind of probiotics, *Bifidobacterium pseudocatenulatum* was found to be positively associated with response to vaccines including bacille Calmette–Guérin and hepatitis B virus vaccines [57].

Upregulation of the superpathway of histidine, purine, and pyrimidine biosynthesis enhanced the production of histidine, purine, and pyrimidine. Histidine has been reported to alleviate the increase in pro-inflammatory factors such as TNF-α and IL-6, induced by a high-fat diet by inhibiting NF-κB signaling and activating the PPARγ pathway [58]. Similar anti-inflammatory mechanisms involving the inhibition of NF-κB signaling on macrophages have been observed in Crohn’s disease models [59]. The acetyl-CoA fermentation to butanoate II pathway produced butyrate, which was consistently reported to exert anti-inflammatory effects [60,61,62]. Butyrate can inhibit the production of pro-inflammatory factors such as interleukin-2 (IL-2) and interferon-γ (IFN-γ), while also accelerating the secretion of anti-inflammatory cytokines like IL-10 and IL-4 in monocytes [63]. Moreover, its classical anti-inflammatory role as an HDAC inhibitor is well recognized for downregulating the function of NF-κB signaling in various immune cells and epithelial cells [64,65,66].

The superpathway of menaquinol-6 biosynthesis, the superpathway of menaquinol-9 biosynthesis and the superpathway of menaquinol-10 biosynthesis produce Vitamin K2, which inhibits the production of pro-inflammatory factors [67] such as inducible nitric oxide synthase (iNOS) and hinders the expression of major histocompatibility complex class II (MHC II) [68]. The superpathway of sulfur amino acid biosynthesis (Saccharomyces cerevisiae), assimilatory sulfate reduction I pathway, and the superpathway of sulfate assimilation and cysteine biosynthesis increase the amount of L-cysteine. This elevation in L-cysteine levels helps alleviate intestinal inflammation induced by LPS. It also downregulates the expression of TNF-α, IL-6, and interleukin 8 (IL-8) by inhibiting p65 nuclear translocation in the NF-κB signaling pathway and increasing NF erythroid 2-related factor 2 (Nrf2) translocation [69].

The upregulation of the thiamine diphosphate salvage II pathway leads to the accumulation of thiamine diphosphate (Vitamin B1), which has been reported to promote humoral immunity and antibody production. Vitamin B1 is essential for T cell-dependent antibody production, primarily involved in anti-viral antibody production, as demonstrated in a lake trout model [70]. Additionally, Vitamin B1 contributes to the maintenance and differentiation of naïve B cell clusters. It aids in the transformation of B cells from producing weak antibody immunoglobulin M (IgM) to generating potent antibody IgA by maintaining the metabolic flux of the TCA cycle. This process is critical for gut IgA responses against orally administered vaccines [71,72]. The superpathway of pyrimidine ribonucleotides de novo biosynthesis can produce cytidine triphosphate (CTP) and uridine 5–triphosphate (UTP). UTP stimulates immune cell migration through purinergic P2Y receptors [73]. The abundance of CTP and UTP may facilitate the translation process of mRNA vaccines and provide enzymes or proteins necessary for antibody generation.

**Figure 3:**
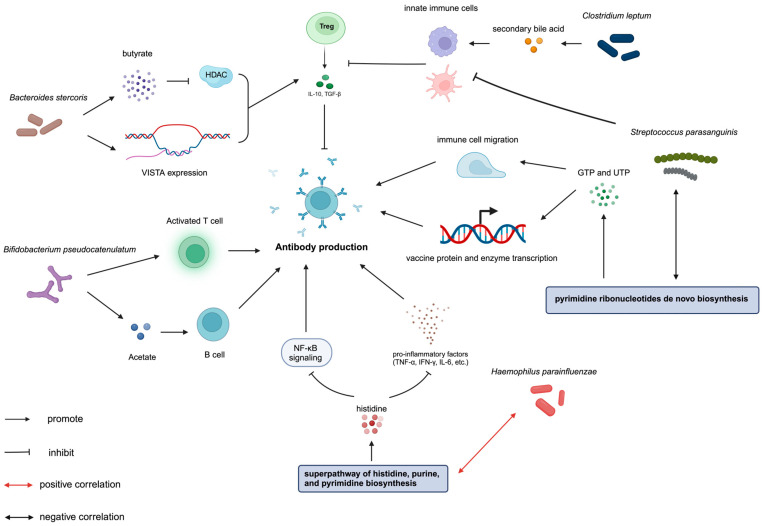
Graphical illustration of the relationship between gut microbiota, metabolic pathways, and BNT162b2 immunogenicity after three doses: insights from our research and prior literature; *Bifidobacterium pseudocatenulatum* can produce acetate to activate B cells as well as increase the proportion of activated T cells. *Bacteroides stercoris* secretes butyrate as HDAC inhibitor and promotes the expression of VISTA gene to prompt Treg cell function. *Streptococcus parasanguinis* enhances the production of IL-10 by facilitating the function of innate immune cells, which activates Treg cells. *Clostridium leptum* can convert primary bile acids to secondary bile acids and facilitate the innate immune response. *Haemophilus parainfluenzae* was positively correlated with the superpathway of histidine, purine, and pyrimidine biosynthesis. Histidine, produced by the superpathway of histidine, purine, and pyrimidine biosynthesis, decreases the secretion of pro-inflammatory factors such as TNF-α, IFN-γ, and IL-6, and inhibits NF-κB signaling. NF-κB signaling plays a key role in the immune reaction to vaccines by mediating the maturation and activation of T cells and B cells. The superpathway of pyrimidine ribonucleotides in de novo biosynthesis can produce CTP and UTP to stimulate immune cell migration and translate mRNA vaccines and enzymes. Abbreviations: HDAC, histone deacetylase; VISTA, V-domain immunoglobulin suppressor of T cell activation; IL-10, interleukin 10; Treg cells, T regulator cells; TNF-α, tumor necrosis factor-α; IFN-γ, Interferon-γ; IL-6, Interleukin 6; CTP, cytidine triphosphate; UTP, uridine 5–triphosphate.

There is a negative correlation between *Streptococcus parasanguinis* and the superpathway of pyrimidine ribonucleotides in de novo biosynthesis. *Haemophilus parainfluenzae* was positively correlated with anti-inflammatory pathways, including the superpathway of histidine, purine, and pyrimidine biosynthesis and the superpathway of purine nucleotides de novo biosynthesis II, while it was negatively correlated with folate transformations II. *Bifidobacterium pseudocatenulatum* was positively correlated with pentose phosphate pathway and negatively correlated with folate transformations II (Figure 3). This correlation suggests a possible mechanism underlying the association between baseline gut microbiota composition and vaccine immunogenicity.

However, there are still several limitations in this study. First, the sample size was relatively small. Second, we did not investigate factors influencing vaccine immunogenicity in terms of metabolomics and proteomics. Demonstrating the correlated variation between bacterial species and metabolites might support the hypothesis that bacteria modulate vaccine immunogenicity by regulating the production of specific metabolites. Therefore, supplementing or depleting specific metabolites, such as SCFAs and tryptophan metabolites, could provide an additional method to enhance vaccine immunogenicity. Moreover, our future studies will require more detailed information, including conditions of anti-inflammatory mediators, additional follow-up intervals, and the vaccination status of other vaccines.

## 5. Conclusions

*Bifidobacterium pseudocatenulatum* and *Clostridium leptum* were associated with a high immune response, while *Bacteroides stercoris*, *Streptococcus parasanguinis* and *Haemophilus parainfluenzae* were associated with a low immune response at one year following three doses of BNT162b2. Metabolic pathways correlated with amnio acid and butyrate synthesis were associated with a high immune response, while pathways related to anti-inflammatory process were associated with a low immune response. These results could establish a basis for future studies to develop innovative strategies that utilize gut microbiota to improve the sustainability of BNT162b2 immunogenicity.

## Figures and Tables

**Table 1:** Baseline characteristics comparison between subjects with low and high immune response at one year following three doses of BNT162b2.

	Whole Cohort N = 125	Low Reponse Group N = 20	High Response Group N = 105	*p*-Value
Age, years, (median (IQR))	52.0 (46.2–59.0)	53.1 (47.5–61.5)	52.0 (46.0–58.3)	0.449
Male (n, %)	43 (34.4%)	11 (55.0%)	32 (30.5%)	0.042
DM or pre-DM (n, %)	50 (40.0%)	10 (50.0%)	40 (38.1%)	0.331
PPI use (n, %) *	18 (17.1%)	2 (10.0%)	16 (15.2%)	0.735

Abbreviation: DM or pre-DM, diabetes mellitus or pre-diabetes mellitus; PPI, proton pump inhibitor; * Usage of ≥14 days within 12 months before first vaccination.

**Table 2:** Univariate and multivariable logistic regression analysis for identifying factors associated with high immune response.

	Univariate Analysis			Multivariable Analysis		
	OR	95% CI	*p* Value	aOR	95% CI	*p* Value
Age ≥ 65 years	1.15	0.18–22.4	0.899	3.43	0.31–107	0.376
Male sex	0.36	0.13–0.95	0.039	0.25	0.06–0.90	0.037
DM or pre-DM	0.62	0.23–1.62	0.322	0.77	0.20–2.90	0.691
PPI use *	1.62	0.41–10.8	0.544	5.08	0.75–64.3	0.142
Smoking history	1.05	0.25–7.18	0.949	4.69	0.67–57.9	0.160
Alcohol history	0.64	0.14–4.55	0.600	0.43	0.05–4.78	0.458
*Bifidobacterium pseudocatenulatum* ^#^	0.53	0.18–1.43	0.226	0.81	0.20–3.17	0.758
*Streptococcus parasanguinis* ^#^	0.35	0.13–0.92	0.035	0.14	0.03–0.60	0.012
*Bacteroides stercoris ^#^*	0.45	0.15–1.22	0.133	0.44	0.11–1.53	0.206
*Clostridium leptum ^#^*	6.91	1.33–127	0.066	12.2	1.73–273	0.036
*Haemophilus parainfluenzae ^#^*	0.38	0.14–1.02	0.053	1.28	0.30–6.39	0.751

* Usage of ≥14 days within 12 months before first vaccination. Unable to generate coefficient and 95% CI for “antibiotic use” due to small number of antibiotic users (*n* = 5). ^#^ High abundance was defined as the top 50% (i.e., above the median). Abbreviations: OR, odds ratio; aOR, adjusted odds ratio; 95% CI, 95% confidence interval; DM or pre-DM, diabetes mellitus or pre-diabetes mellitus; PPI, proton pump inhibitors.

## Data Availability

The data presented in this study are available upon request from the corresponding author due to confidentiality issues.

## Supplementary Materials

The following supporting information can be downloaded at: https://www.mdpi.com/article/10.3390/vaccines12080916/s1, Figure S1. Flowchart of the study cohort; Figure S2. Comparison of microbiota diversity between low and high immune response groups; Figure S3. Baseline bacterial species and metabolic pathways enriched in low and high immune response group after excluding subjects with prior antibiotic use; Figure S4. Comparison of gut microbiota composition at baseline and one year following three doses of BNT162b2; Figure S5. Comparison of gut microbiota diversity between baseline and one year following three doses of BNT162b2; Figure S6. Comparison of the relative abundance of putative bacterial species between baseline and one year following three doses of BNT162b2; Figure S7. Baseline metabolic pathways enriched in low and high immune response groups; Table S1. Baseline characteristics comparison between subjects with low and high immune response at one year following three doses of BNT162b2 after excluding subjects with prior use of antibiotics; Table S2. Summary of the identified metabolic pathways.
