# Supplementary material for: Baseline Gut Microbiota Was Associated with Long-Term Immune Response at One Year Following Three Doses of BNT162b2
Source: Vaccines (Basel). 2024 Aug 14;12(8):916. doi: 10.3390/vaccines12080916 (PMC11359560; doi:10.3390/vaccines12080916)
Supplement: Supplementary file 1 [file vaccines-12-00916-s001.zip › vaccines-3099797-supplementary.pdf]

**Figure S1. Flowchart of the study cohort**

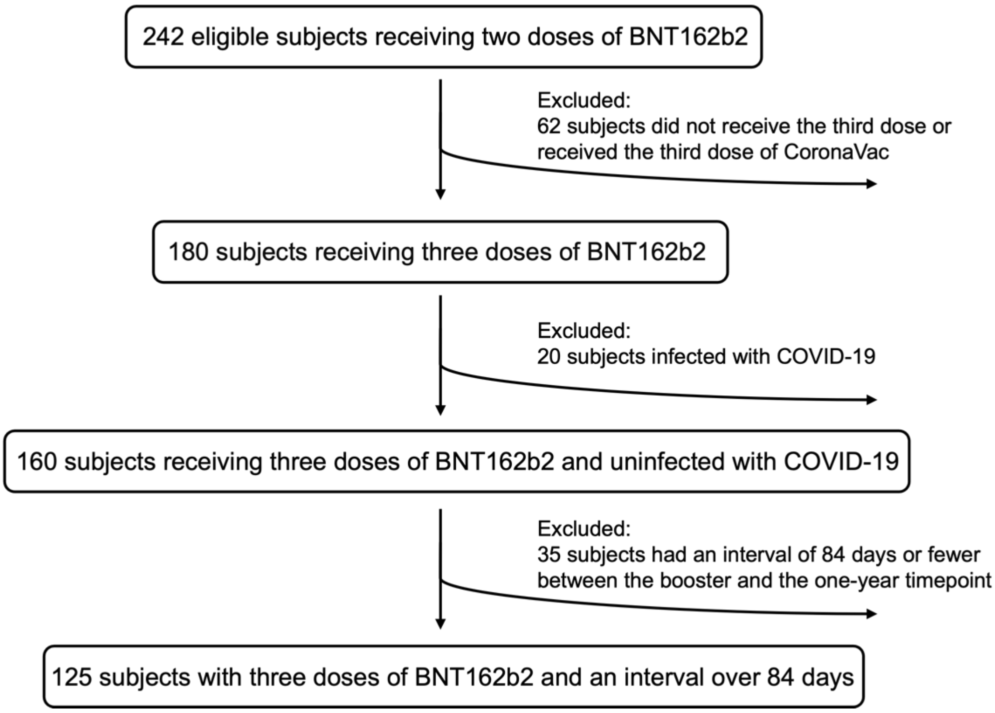

**Figure S2. Comparison of gut microbiota diversity between low and high immune response groups**

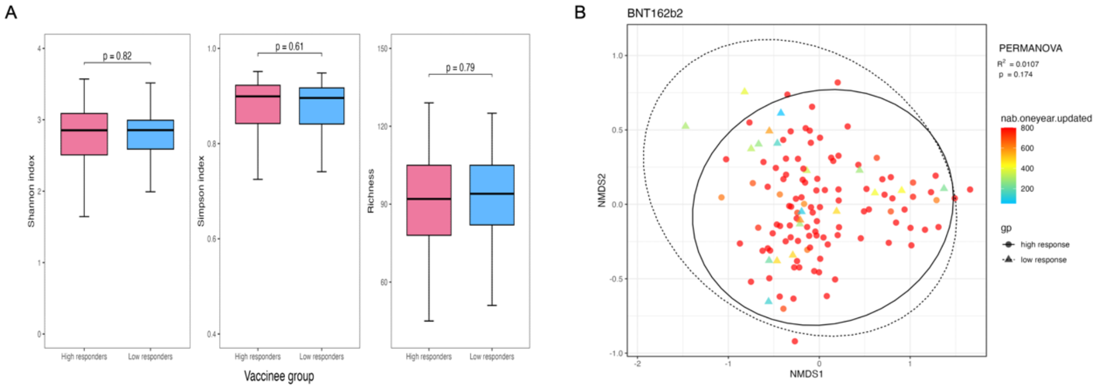


A. Comparison of alpha diversity between low and high immune response groups in terms of Shannon index, Simpson indices, species richness.

B. Comparison of beta diversity between low and high immune response group

**Figure S3. Baseline bacterial species and metabolic pathways enriched in low and high immune response group after excluding subjects with prior antibiotic use**

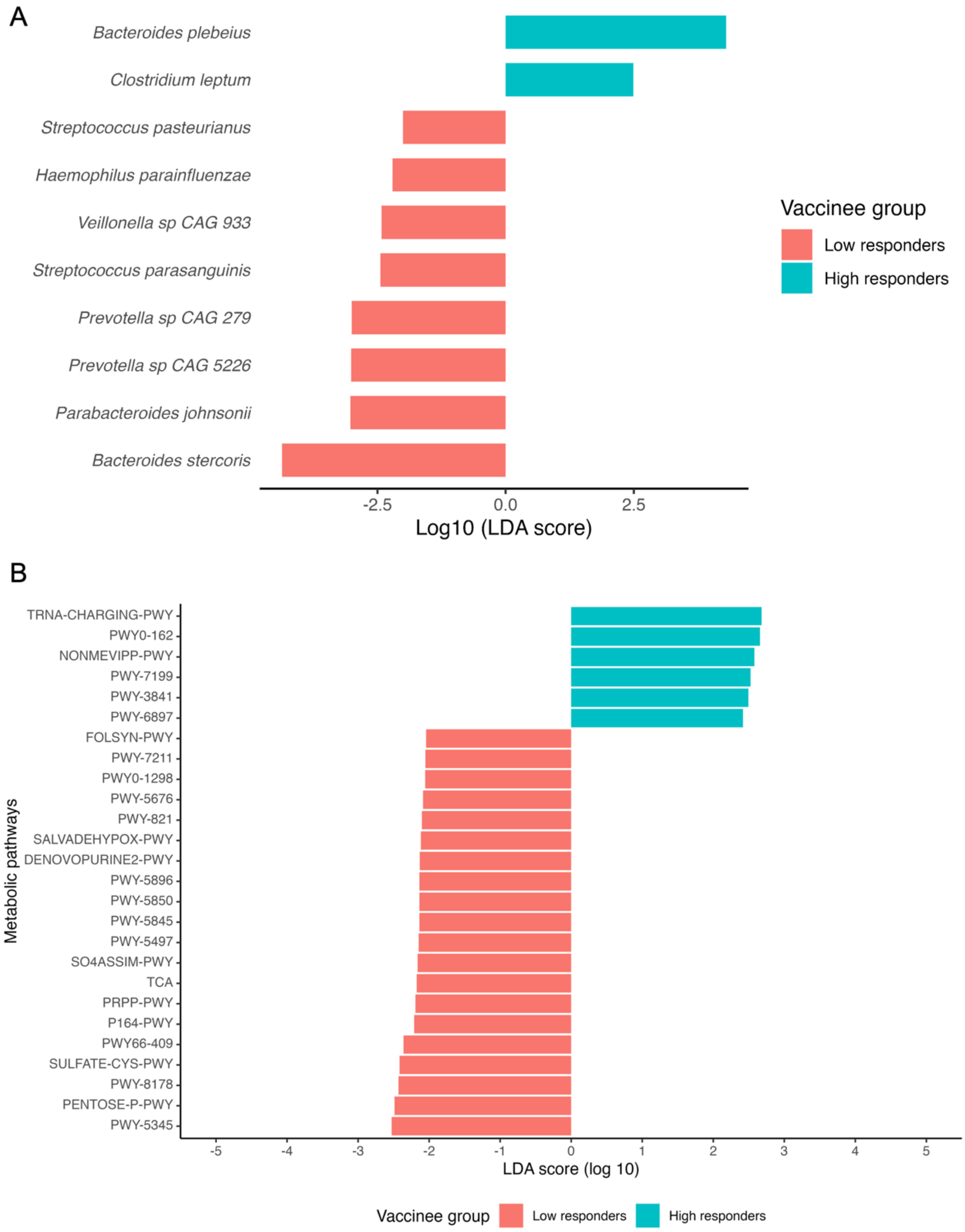


A. Bacterial species enriched in low and high immune response groups identified by LEfSe analysis, subjects with antibiotics use history were excluded.

B. Metabolic pathways enriched in low and high immune response groups identified by LEfSe analysis, subjects with antibiotics use history were excluded.

**Figure S4. Comparison of gut microbiota composition at baseline and one year following three doses of BNT162b2**

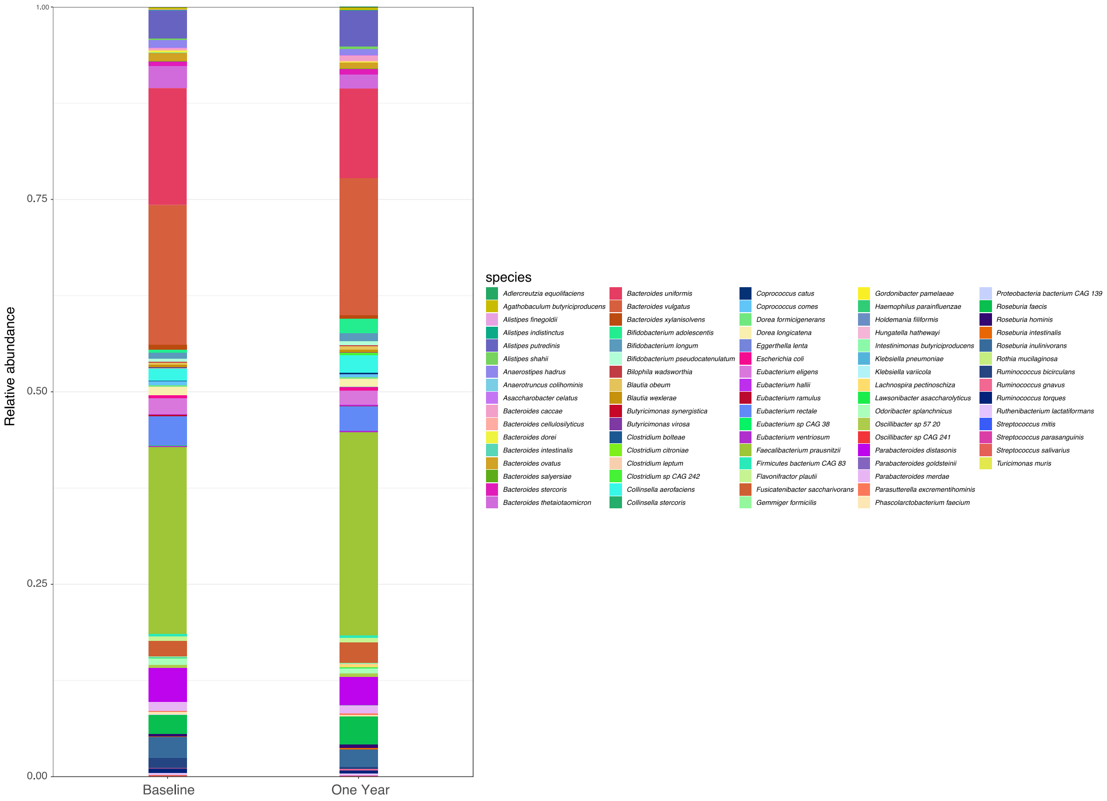


Profile of bacterial species with a relative abundance greater than 0 at baseline and one-year timepoint.

**Figure S5. Comparison of gut microbiota diversity between baseline and one year following three doses of BNT162b2**

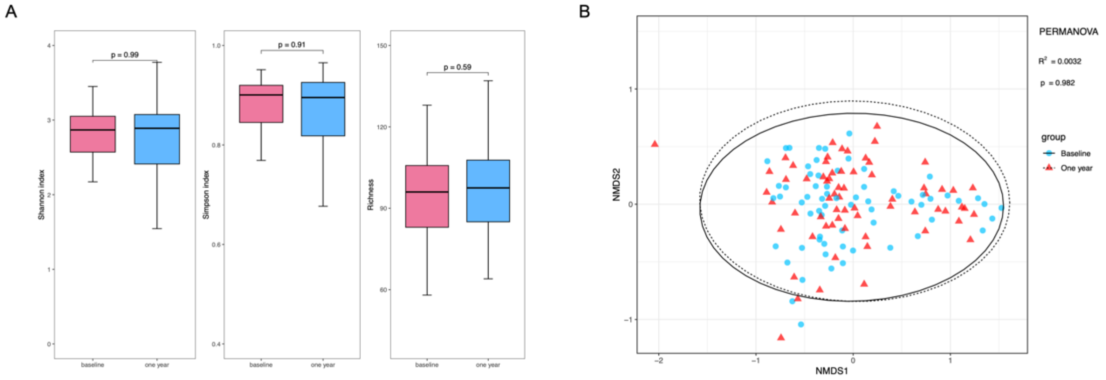


A. Comparison of alpha diversity of gut microbiota between baseline and one-year timepoint in terms of Shannon index, Simpson indices, species richness.

B. Comparison of beta diversity of gut microbiota between baseline and one-year timepoint.

**Figure S6. Comparison of the relative abundance of putative bacterial species between baseline and one year following three doses of BNT162b2**

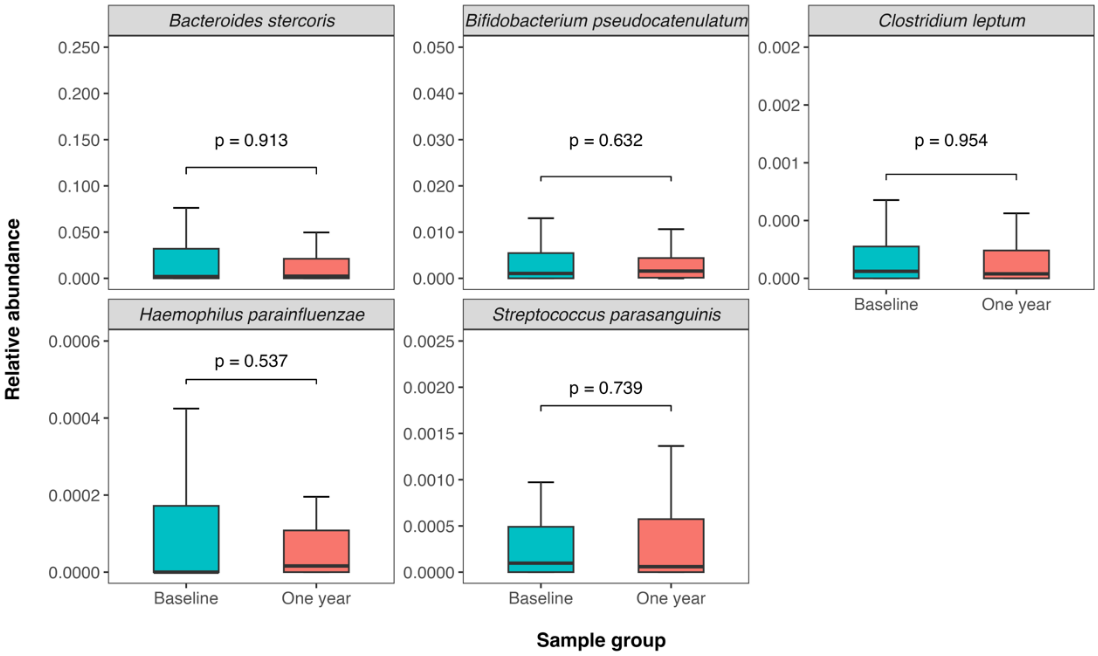

**Figure S7. Baseline metabolic pathways enriched in low and high immune response groups**

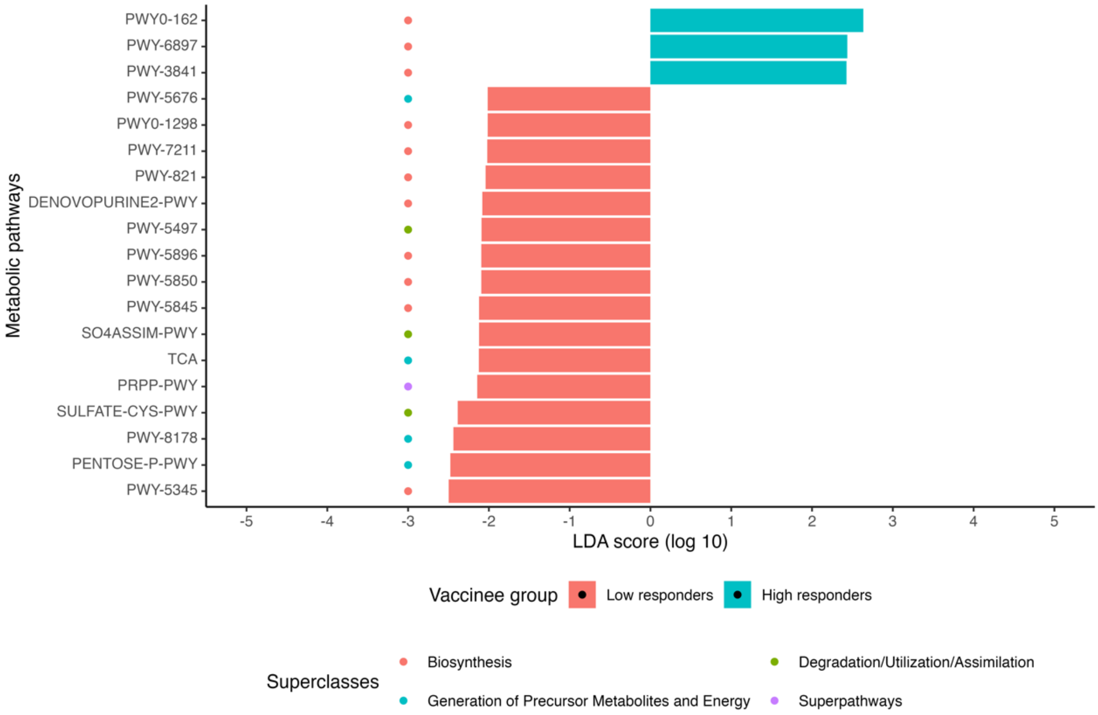


Metabolic pathways enriched in low and high immune response groups identified by LEfSe analysis.

**Table S1. Baseline characteristics comparison between subjects with low and high immune response at one year following three doses of BNT162b2 after excluding subjects with prior use of antibiotics**

| | **Whole cohort N=120** | **Low reponse group N=20** | **High response group N=100** | **p-value** |
|---|---|---|---|---|
| Age,years, (median(IQR)) | 52.0 (46.2-59.0) | 53.1 (47.5-61.5) | 52.0 (45.9-58.4) | 0.412 |
| Male (n,%) | 40（33.3%） | 11 (55.0%) | 29 (29.0%) | 0.036 |
| DM or pre-DM (n,%) | 48 (40.0%) | 10 (50.0%) | 38 (38.0%) | 0.330 |
| PPI use (n,%)* | 15 (12.5%) | 2 (10.0%) | 13 (13.0%) | 1.000 |

Abbreviation: DM or pre-DM, diabetes mellitus or pre-diabetes mellitus; PPI, proton pump inhibitor.

* Usage of ≥14 days within 12 months before first vaccination.

**Table S2. Summary of the identified metabolic pathways**

| Abbreviation | Full name | Superclass | Vaccinee group | LDA score | p-value |
|---|---|---|---|---|---|
| PWY-3841 | folate transformations II | Biosynthesis | high response | 2.43 | 0.039 |
| PWY-6897 | thiamine diphosphate salvage II | Biosynthesis | high response | 2.44 | 0.022 |
| PWY0-162 | superpathway of pyrimidine ribonucleotides de novo biosynthesis | Biosynthesis | high response | 2.63 | 0.001 |
| SULFATE-CYS-PWY | superpathway of sulfate assimilation and cysteine biosynthesis | Degradation/Utilization/Assimilation | Low response | 2.39 | 0.025 |
| TCA | TCA cycle I (prokaryotic) | Generation of Precursor Metabolites and Energy | Low response | 2.12 | 0.027 |
| SO4ASSIM-PWY | assimilatory sulfate reduction I | Degradation/Utilization/Assimilation | Low response | 2.12 | 0.030 |
| PWY0-1298 | superpathway of pyrimidine deoxyribonucleosides degradation | Biosynthesis | Low response | 2.02 | 0.005 |
| PWY-821 | superpathway of sulfur amino acid biosynthesis (Saccharomyces cerevisiae) | Biosynthesis | Low response | 2.04 | 0.023 |
| PWY-8178 | pentose phosphate pathway (non-oxidative branch) II | Generation of Precursor Metabolites and Energy | Low response | 2.44 | 0.039 |
| PWY-7211 | superpathway of pyrimidine deoxyribonucleotides de novo biosynthesis | Biosynthesis | Low response | 2.02 | 0.033 |
| DENOVOPURINE2-PWY | superpathway of purine nucleotides de novo biosynthesis II | Biosynthesis | Low response | 2.08 | 0.006 |
| PWY-5896 | superpathway of menaquinol-10 biosynthesis | Biosynthesis | Low response | 2.09 | 0.013 |
| PWY-5850 | superpathway of menaquinol-6 biosynthesis | Biosynthesis | Low response | 2.09 | 0.013 |
| PWY-5845 | superpathway of menaquinol-9 biosynthesis | Biosynthesis | Low response | 2.12 | 0.039 |
| PWY-5676 | acetyl-CoA fermentation to butanoate II | Generation of Precursor Metabolites and Energy | Low response | 2.01 | 0.034 |
| PWY-5497 | purine nucleobases degradation II (anaerobic) | Degradation/Utilization/Assimilation | Low response | 2.09 | 0.026 |
| PWY-5345 | superpathway of L-methionine biosynthesis (by sulfhydrylation) | Biosynthesis | Low response | 2.50 | 0.015 |
| PENTOSE−P−PWY | pentose phosphate pathway | Generation of Precursor Metabolites and Energy | Low response | 2.48 | 0.046 |
| PRPP−PWY | superpathway of histidine, purine, and pyrimidine biosynthesis | Superpathways | Low response | 2.14 | 0.006 |
